# Supplementary material for: Walk Your Talk: Real-World Adherence to Guidelines on the Use of MRI in Multiple Sclerosis
Source: Diagnostics (Basel). 2021 Jul 21;11(8):1310. doi: 10.3390/diagnostics11081310 (PMC8394408; doi:10.3390/diagnostics11081310)
Supplement: Supplementary file 1 [file diagnostics-11-01310-s001.zip › diagnostics-1277579-supplementary.pdf]

Real-life MR protocols for Multiple Sclerosis diagnosis and follow-up

Fields marked with * are mandatory.

1 Before you start

This survey in not designed to assess the theoretical knowledge about recommended standardized MR protocols for Multiple Sclerosis, but rather to get a picture of what happens in clinical practice, so please answer truthfully! Also, refer only to clinical exams and not to exams acquired for research purposes.

2 Something about yourself

* 1) How long ago did you earn your degree in radiology?
  - still in training
  - < 5 years ago
  - between 5 and 10 years ago
  - > 10 years ago

* 2) How many exams for suspected/confirmed Multiple Sclerosis do you see in a month?
  - 0-5
  - 5-10
  - 10-20
  - >20

* 3) Where do you see them?
  - hospital
  - university hospital
  - other (e.g. private diagnostic clinics)

* please specify

* 4) In which Italian region do you work?

  - Abruzzo
  - Basilicata
  - Calabria
  - Campania
  - Emilia-Romagna

- Friuli venezia Giulia
- Lazio
- Liguria
- Lombardia
- Marche
- Molise
- Piemonte
- Puglia
- Sardegna
- Sicilia
- Toscana
- Trentino-Alto Adige
- Umbria
- Valle d'Aosta
- Veneto

3 MRI protocol

In the following questions we are going to ask you information about the protocol that you use in your clinical practice

4 Field strength

* 5) At which magnetic field strength do you usually acquire MS patients?

- <1.5T
- 1.5T
- 3T

5 MS diagnostic brain exam

The following questions specifically refer to the sequences that you usually acquire for a MS diagnostic brain exam in your clinical practice.

6 T1-weighted sequences - Diagnostic exam

* Which of these sequences do you usually acquire for a MS diagnostic brain exam? (check all that apply)

- 2D SE T1-weighted sequence (pre-Gad)
- 3D TSE T1-weighted sequence (pre-Gad)
- 3D GrE T1-weighted sequence (pre-Gad)
- 2D SE T1-weighted sequence (post-Gad)
- 3D TSE T1-weighted sequence (post-Gad)
- 3D GrE T1-weighted sequence (post-Gad)
- other (please specify)

* please specify

* slice thickness for 2D SE T1-weighted sequence (pre-Gad)
  - < or = 3 mm
  - > 3 mm

* gap for 2D SE T1-weighted sequence (pre-Gad)
  - yes
  - no

* voxel size for 3D TSE T1-weighted sequence (pre-Gad)
  - < 1 mm isotropic
  - = 1 mm isotropic
  - other (e.g. 0.7x0.7x1.5 mm)

* voxel size for 3D GrE T1-weighted sequence (pre-Gad)

- < 1 mm isotropic
- = 1 mm isotropic
- other (e.g. 0.7x0.7x1.5 mm)

* slice thickness for 2D SE T1-weighted sequence (post-Gad)

- < or = 3 mm
- > 3 mm

* gap for 2D SE T1-weighted sequence (post-Gad)

- yes
- no

* delay for 2D SE T1-weighted sequence (post-Gad)

- < or = 5 minutes
- > 5 minutes

* voxel size for 3D TSE T1-weighted sequence (post-Gad)

- < 1 mm isotropic
- = 1 mm isotropic
- other (e.g. 0.7x0.7x1.5 mm)

* delay for 3D TSE T1-weighted sequence (post-Gad)

- < or = 5 minutes
- > 5 minutes

* voxel size for 3D GrE T1-weighted sequence (post-Gad)

- < 1 mm isotropic
- = 1 mm isotropic
- other (e.g. 0.7x0.7x1.5 mm)

* delay for 3D GrE T1-weighted sequence (post-Gad)

- < or = 5 minutes
- > 5 minutes

7 T2-weighted sequences - Diagnostic exam

* Which of these sequences do you usually acquire for a MS diagnostic brain exam? (check all that apply)

- 2D Dual Echo sequence
- 2D T2-weighted sequence
- 3D T2-weighted sequence

- ☐ 2D FLAIR sequence
- ☐ 3D FLAIR sequence
- ☐ other (please specify)

* please specify

[ ]

* slice thickness for 2D Dual Echo sequence
  - ○ < or = 3 mm
  - ○ > 3 mm

* gap for 2D Dual Echo sequence
  - ○ yes
  - ○ no

* slice thickness for 2D T2-weighted sequence
  - ○ < or = 3 mm
  - ○ > 3 mm

* gap for 2D T2-weighted sequence
  - ○ yes
  - ○ no

* voxel size for 3D T2-weighted sequence
  - ○ < 1 mm isotropic
  - ○ = 1 mm isotropic
  - ○ other (e.g. 0.7x0.7x1.5 mm)

* slice thickness for 2D FLAIR sequence
  - ○ < or = 3 mm
  - ○ > 3 mm

* gap for 2D FLAIR sequence
  - ○ yes
  - ○ no

* voxel size for 3D FLAIR sequence
  - ○ < 1 mm isotropic
  - ○ = 1 mm isotropic
  - ○ other (e.g. 0.7x0.7x1.5 mm)

8 Cortical lesion sequences - Diagnostic exam

* Which of these sequences do you usually acquire for a MS diagnostic brain exam? (check all that apply)

- ☐ 2D DIR sequence
- ☐ 3D DIR sequence
- ☐ 2D PSIR sequence
- ☐ 3D PSIR sequence
- ☐ none
- ☐ other (please specify)

* please specify

* slice thickness for 2D DIR sequence

- ○ < or = 3 mm
- ○ > 3 mm

* gap for 2D DIR sequence

- ○ yes
- ○ no

* voxel size for 3D DIR sequence

- ○ < 1 mm isotropic
- ○ = 1 mm isotropic
- ○ other (e.g. 0.7x0.7x1.5 mm)

* slice thickness for 2D PSIR sequence

- ○ < or = 3 mm
- ○ > 3 mm

* gap for 2D PSIR sequence

- ○ yes
- ○ no

* voxel size for 3D PSIR sequence

- ○ < 1 mm isotropic
- ○ = 1 mm isotropic
- ○ other (e.g. 0.7x0.7x1.5 mm)

9 Optic nerve sequences - Diagnostic exam

* Which of these sequences do you usually acquire for a MS diagnostic brain exam? (check all that apply)

- ☐ Fat-suppressed T2-weigthed (or STIR) for optic nerves
- ☐ Fat-suppressed T1-weighted, post-Gad, for optic nerves
- ☐ none

- [ ] other (please specify)

* please specify

* slice thickness for Fat-suppressed T2-weigthed (or STIR) for optic nerves

- ( ) < or = 3 mm
- ( ) > 3 mm

* gap for Fat-suppressed T2-weigthed (or STIR) for optic nerves

- ( ) yes
- ( ) no

* slice thickness for Fat-suppressed T1-weighted, post-Gad, for optic nerves

- ( ) < or = 3 mm
- ( ) > 3 mm

* gap for Fat-suppressed T1-weighted, post-Gad, for optic nerves

- ( ) yes
- ( ) no

10 DWI & T2*-weighted sequences - Diagnostic exam

* Which of these sequences do you usually acquire for a MS diagnostic brain exam? (check all that apply)

- [ ] DWI
- [ ] T2*-weighted GrE sequence
- [ ] 2D SWI sequence
- [ ] 3D SWI sequence
- [ ] none
- [ ] other (please specify)

* please specify

* slice thickness for T2*-weighted GrE sequence

- ( ) < or = 3 mm
- ( ) > 3 mm

* gap for T2*-weighted GrE sequence

- ( ) yes
- ( ) no

* slice thickness for 2D SWI sequence

- < or = 3 mm
- > 3 mm

* gap for 2D SWI sequence

- yes
- no

* voxel size for 3D SWI sequence

- < 1 mm isotropic
- = 1 mm isotropic
- other (e.g. 0.7x0.7x1.5 mm)

11 MS diagnostic spine exam

the following questions specifically refer to the sequences that you usually acquire for a MS diagnostic spine exam in your clinical practice.

12 T2-weighted sequences - Diagnostic exam

* Which of these sequences do you usually acquire for a diagnostic spine exam?

- TSE T2-weighted sequence
- PD-weighted sequence
- STIR T2-weighted sequence
- other (please specify)

* please specify

* in which plane do you acquire the TSE T2-weighted sequence?

- sagittal
- axial

* slice thickness of sagittal TSE T2-weighted sequence

- < or = 3 mm
- > 3 mm

* gap of sagittal TSE T2-weighted sequence

- yes
- no

* in which plane do you acquire the PD-weighted sequence?

- sagittal

- [ ] axial

* slice thickness of sagittal PD-weighted sequence

- ( ) < or = 3 mm
- ( ) > 3 mm

* gap of sagittal PD-weighted sequence

- ( ) yes
- ( ) no

* in which plane do you acquire the STIR T2-weighted sequence?

- [ ] sagittal
- [ ] axial

* slice thickness of sagittal STIR T2-weighted sequence

- ( ) < or = 3 mm
- ( ) > 3 mm

* gap of sagittal STIR T2-weighted sequence

- ( ) yes
- ( ) no

13 T1-weighted and inversion recovery - Diagnostic exam

* Which of these sequences do you usually acquire for a diagnostic spine exam?

- [ ] PSIR sequence
- [ ] 2D TSE T1-weighted sequence (pre-Gad)
- [ ] 3D GrE T1-weighted sequence (pre-Gad)
- [ ] 2D TSE T1-weighted sequence (post-Gad)
- [ ] 3D GrE T1-weighted sequence (post-Gad)
- [ ] other (please specify)

* please specify

* in which plane do you acquire the PSIR sequence?

- [ ] sagittal
- [ ] axial

* slice thickness of sagittal PSIR sequence

- ( ) < or = 3 mm
- ( ) > 3 mm

* gap of sagittal PSIR sequence

- yes
- no

* in which plane do you acquire the 2D TSE T1-weighted sequence (pre-Gad)?

- sagittal
- axial

* slice thickness of sagittal 2D TSE T1-weighted sequence (pre-Gad)

- < or = 3 mm
- > 3 mm

* gap of sagittal 2D TSE T1-weighted sequence (pre-Gad)

- yes
- no

* in which plane do you acquire the 3D GrE T1-weighted sequence (pre-Gad)?

- sagittal
- axial

* slice thickness of 3D GrE T1-weighted sequence (pre-Gad)?

- < or = 3 mm
- > 3 mm

* gap of 3D GrE T1-weighted sequence (pre-Gad)?

- yes
- no

* in which plane do you acquire the 2D TSE T1-weighted sequence (post-Gad)

- sagittal
- axial

* slice thickness of sagittal 2D TSE T1-weighted sequence (post-Gad)

- < or = 3 mm
- > 3 mm

* gap of sagittal 2D TSE T1-weighted sequence (post-Gad)

- yes
- no

* delay for 2D TSE T1-weighted sequence (post-Gad)

- < or = 5 minutes
- > 5 minutes

* in which plane do you acquire the 3D GrE T1-weighted sequence (post-Gad)?

- sagittal

- [ ] axial

* slice thickness of 3D GrE T1-weighted sequence (post-Gad)?

- ( ) < or = 3 mm
- ( ) > 3 mm

* gap of 3D GrE T1-weighted sequence (post-Gad)?

- ( ) yes
- ( ) no

* delay for 3D GrE T1-weighted sequence (post-Gad)

- ( ) < or = 5 minutes
- ( ) > 5 minutes

14 MS brain follow-up exam

The following questions specifically refer to the sequences that you usually acquire for a MS brain follow-up exam in your clinical practice.

15 T1-weighted sequences - Follow up exam

* Which of these sequences do you usually acquire for a MS brain follow-up exam? (check all that apply)

- [ ] 2D SE T1-weighted sequence (pre-Gad)
- [ ] 3D TSE T1-weighted sequence (pre-Gad)
- [ ] 3D GrE T1-weighted sequence (pre-Gad)
- [ ] 2D SE T1-weighted sequence (post-Gad)
- [ ] 3D TSE T1-weighted sequence (post-Gad)
- [ ] 3D GrE T1-weighted sequence (post-Gad)
- [ ] other (please specify)

* please specify

* slice thickness for 2D SE T1-weighted sequence (pre-Gad)

- ○ < or = 3 mm
- ○ > 3 mm

* gap for 2D SE T1-weighted sequence (pre-Gad)

- ○ yes
- ○ no

* voxel size for 3D TSE T1-weighted sequence (pre-Gad)

- ○ < 1 mm isotropic
- ○ = 1 mm isotropic
- ○ other (e.g. 0.7x0.7x1.5 mm)

* voxel size for 3D GrE T1-weighted sequence (pre-Gad)

- ○ < 1 mm isotropic
- ○ = 1 mm isotropic
- ○ other (e.g. 0.7x0.7x1.5 mm)

* slice thickness for 2D SE T1-weighted sequence (post-Gad)

- ○ < or = 3 mm
- ○ > 3 mm

* gap for 2D SE T1-weighted sequence (post-Gad)

- ○ yes
- ○ no

* delay for 2D SE T1-weighted sequence (post-Gad)

- ○ < or = 5 minutes
- ○ > 5 minutes

* voxel size for 3D TSE T1-weighted sequence (post-Gad)

- ○ < 1 mm isotropic

- = 1 mm isotropic
- other (e.g. 0.7x0.7x1.5 mm)

* delay for 3D TSE T1-weighted sequence (post-Gad)

- < or = 5 minutes
- > 5 minutes

* voxel size for 3D GrE T1-weighted sequence (post-Gad)

- < 1 mm isotropic
- = 1 mm isotropic
- other (e.g. 0.7x0.7x1.5 mm)

* delay for 3D GrE T1-weighted sequence (post-Gad)

- < or = 5 minutes
- > 5 minutes

16 T2-weighted sequences - Follow up exam

* Which of these sequences do you usually acquire for a MS brain <u>follow-up</u> exam? (check all that apply)

- [ ] 2D Dual Echo sequence
- [ ] 2D T2-weighted sequence
- [ ] 3D T2-weighted sequence
- [ ] 2D FLAIR sequence
- [ ] 3D FLAIR sequence
- [ ] other (please specify)

* please specify

* slice thickness for 2D Dual Echo sequence

- < or = 3 mm
- > 3 mm

* gap for 2D Dual Echo sequence

- yes
- no

* slice thickness for 2D T2-weighted sequence

- < or = 3 mm
- > 3 mm

* gap for 2D T2-weighted sequence

- yes

- no

* voxel size for 3D T2-weighted sequence
- < 1 mm isotropic
- = 1 mm isotropic
- other (e.g. 0.7x0.7x1.5 mm)

* slice thickness for 2D FLAIR sequence
- < or = 3 mm
- > 3 mm

* gap for 2D FLAIR sequence
- yes
- no

* voxel size for 3D FLAIR sequence
- < 1 mm isotropic
- = 1 mm isotropic
- other (e.g. 0.7x0.7x1.5 mm)

17 Cortical lesion sequences - Follow up exam

* Which of these sequences do you usually acquire for a MS brain follow-up exam? (check all that apply)
- 2D DIR sequence
- 3D DIR sequence
- 2D PSIR sequence
- 3D PSIR sequence
- none
- other (please specify)

* please specify

* slice thickness for 2D DIR sequence
- < or = 3 mm
- > 3 mm

* gap for 2D DIR sequence
- yes
- no

* voxel size for 3D DIR sequence
- < 1 mm isotropic

- = 1 mm isotropic
- other (e.g. 0.7x0.7x1.5 mm)

* slice thickness for 2D PSIR sequence

- < or = 3 mm
- > 3 mm

* gap for 2D PSIR sequence

- yes
- no

* voxel size for 3D PSIR sequence

- < 1 mm isotropic
- = 1 mm isotropic
- other (e.g. 0.7x0.7x1.5 mm)

18 Optic nerve sequences - Follow up exam

* Which of these sequences do you usually acquire for a MS brain follow-up exam? (check all that apply)

- [ ] Fat-suppressed T2-weigthed (or STIR) for optic nerves
- [ ] Fat-suppressed T1-weighted, post-Gad, for optic nerves
- [ ] none
- [ ] other (please specify)

* please specify

* slice thickness for Fat-suppressed T2-weigthed (or STIR) for optic nerves

- < or = 3 mm
- > 3 mm

* gap for Fat-suppressed T2-weigthed (or STIR) for optic nerves

- yes
- no

* slice thickness for Fat-suppressed T1-weighted, post-Gad, for optic nerves

- < or = 3 mm
- > 3 mm

* gap for Fat-suppressed T1-weighted, post-Gad, for optic nerves

- yes
- no

19 DWI & T2*-weighted sequences - Follow up exam

* Which of these sequences do you usually acquire for a MS brain follow-up exam? (check all that apply)

- ☐ DWI
- ☐ T2*-weighted GrE sequence
- ☐ 2D SWI sequence
- ☐ 3D SWI sequence
- ☐ none
- ☐ other (please specify)

* please specify

* slice thickness for T2*-weighted GrE sequence

- ○ < or = 3 mm
- ○ > 3 mm

* gap for T2*-weighted GrE sequence

- ○ yes
- ○ no

* slice thickness for 2D SWI sequence

- ○ < or = 3 mm
- ○ > 3 mm

* gap for 2D SWI sequence

- ○ yes
- ○ no

* voxel size for 3D SWI sequence

- ○ < 1 mm isotropic
- ○ = 1 mm isotropic
- ○ other (e.g. 0.7x0.7x1.5 mm)

20 MS spine follow-up exam

the following questions specifically refer to the sequences that you usually acquire for a MS spine follow-up exam in your clinical practice.

21 T2-weighted sequences - Follow up exam

* Which of these sequences do you usually acquire for a spine follow-up exam?

- [ ] TSE T2-weighted sequence
- [ ] PD-weighted sequence
- [ ] STIR T2-weighted sequence
- [ ] other (please specify)

* please specify

* in which plane do you acquire the TSE T2-weighted sequence?

- [ ] sagittal
- [ ] axial

* slice thickness of sagittal TSE T2-weighted sequence

- ○ < or = 3 mm
- ○ > 3 mm

* gap of sagittal TSE T2-weighted sequence

- ○ yes
- ○ no

* in which plane do you acquire the PD-weighted sequence?

- [ ] sagittal
- [ ] axial

* slice thickness of sagittal PD-weighted sequence

- ○ < or = 3 mm
- ○ > 3 mm

* gap of sagittal PD-weighted sequence

- ○ yes
- ○ no

* in which plane do you acquire the STIR T2-weighted sequence?

- [ ] sagittal
- [ ] axial

* slice thickness of sagittal STIR T2-weighted sequence

- ○ < or = 3 mm
- ○ > 3 mm

* gap of sagittal STIR T2-weighted sequence

- ○ yes
- ○ no

22 T1-weighted and inversion recovery - Follow up exam

* Which of these sequences do you usually acquire for a spine follow-up exam?

- ☐ PSIR sequence
- ☐ 2D TSE T1-weighted sequence (pre-Gad)
- ☐ 3D GrE T1-weighted sequence (pre-Gad)
- ☐ 2D TSE T1-weighted sequence (post-Gad)
- ☐ 3D GrE T1-weighted sequence (post-Gad)
- ☐ other (please specify)

* please specify

* in which plane do you acquire the PSIR sequence?

- ☐ sagittal
- ☐ axial

* slice thickness of sagittal PSIR sequence

- ○ < or = 3 mm
- ○ > 3 mm

* gap of sagittal PSIR sequence

- ○ yes
- ○ no

* in which plane do you acquire the 2D TSE T1-weighted sequence (pre-Gad)?

- ☐ sagittal
- ☐ axial

* slice thickness of sagittal 2D TSE T1-weighted sequence (pre-Gad)

- ○ < or = 3 mm
- ○ > 3 mm

* gap of sagittal 2D TSE T1-weighted sequence (pre-Gad)

- ○ yes
- ○ no

* in which plane do you acquire 3D GrE T1-weighted sequence (pre-Gad)

- ☐ sagittal
- ☐ axial

* slice thickness of 3D GrE T1-weighted sequence (pre-Gad)

- ○ < or = 3 mm
- ○ > 3 mm

* gap of 3D GrE T1-weighted sequence (pre-Gad)

- ○ yes
- ○ no

* in which plane do you acquire the 2D TSE T1-weighted sequence (post-Gad)

- [ ] sagittal
- [ ] axial

* slice thickness of sagittal 2D TSE T1-weighted sequence (post-Gad)

- ( ) < or = 3 mm

- > 3 mm

* gap of sagittal 2D TSE T1-weighted sequence (post-Gad)

- yes
- no

* delay for 2D TSE T1-weighted sequence (post-Gad)

- < or = 5 minutes
- > 5 minutes

* in which plane do you acquire 3D GrE T1-weighted sequence (post-Gad)

- sagittal
- axial

* slice thickness of 3D GrE T1-weighted sequence (post-Gad)

- < or = 3 mm
- > 3 mm

* gap of 3D GrE T1-weighted sequence (post-Gad)

- yes
- no

* delay for 3D GrE T1-weighted sequence (post-Gad)

- < or = 5 minutes
- > 5 minutes

23 Report

* Do you use a structured or descriptive report?

- structured
- descriptive

* Which of these information are included in your baseline report? (check all that apply)

- anatomical area covered
- field strength
- slice thickness
- type and dose of contrast agent used
- type of sequences performed
- lesion number
- lesion size and or shape
- lesion location
- Gad enhancing lesions
- atrophy (qualitative assessment)
- cortical lesions
- black holes
- involvement of the optic nerve

* for lesion number

- absolute number
- range (e.g. <10, between 10 and 20, >20)

* for lesion location

- all locations
- typical location according to diagnostic criteria

* Which of these findings/features are included in your follow-up report? (check all that apply)

- [ ] anatomical area covered
- [ ] field strength
- [ ] slice thickness
- [ ] type and dose of contrast agent used
- [ ] type of sequences performed
- [ ] lesion number
- [ ] for previously described lesions: lesion size and/or shape
- [ ] for previously described lesions: lesion location
- [ ] Gad enhancing lesions
- [ ] new T2-weighted lesions
- [ ] enlarging T2-weighted lesions
- [ ] atrophy (qualitative assessment)
- [ ] cortical lesions
- [ ] black holes
- [ ] involvement of the optic nerve

* for previously described lesions: lesion number

- ○ absolute number
- ○ range (e.g. <10, between 10 and 20, >20)

* for previously described lesions: lesion location

- ○ all locations
- ○ typical location according to diagnostic criteria

* In your conclusion (radiological interpretation) do you formally refer to the 2017 revisions of the McDonald criteria?

- ○ yes
- ○ no

24 Practicalities

* On average, how long does it take to write a full report for a MS diagnostic exam?

- ○ less than 15 minutes
- ○ between 15 and 30 minutes
- ○ more than 30 minutes

* On average, how long does it take to write a full report for a MS follow-up exam?

- ○ less than 15 minutes
- ○ between 15 and 30 minutes
- ○ more than 30 minutes

* At your Institution, which type of contrast agent do you usually administer to MS patients?

- ○ macrocyclic only
- ○ linear only
- ○ either macrocyclic or linear

25 The future

* Among the following pathophysiology-based MR markers, which one do you think it is ready for implementation in clinical practice? (check all that apply)

- [ ] central vein sign
- [ ] subpial demyelination
- [ ] smoldering/slowly expanding lesion
- [ ] quantitative evaluation of brain atrophy
- [ ] quantitative evaluation of spine atrophy
- [ ] none

* why? (check all that apply)

- [ ] technical issues related to sequence availability
- [ ] acquisition time constraints
- [ ] reporting time constraints
- [ ] lack of specific training for identification of the proposed markers
- [ ] none of the proposed marker is a good expression of disease pathophysiology

26 Additional comments

Please use this space to add any comments to topics that have not been included, or have been only partly covered, in this survey
